# Evaluating Spatial Hearing Using a Dual-Task Approach in a Virtual-Acoustics Environment

**DOI:** 10.3389/fnins.2022.787153

**Published:** 2022-03-08

**Authors:** Marina Salorio-Corbetto, Ben Williges, Wiebke Lamping, Lorenzo Picinali, Deborah Vickers

**Affiliations:** ^1^SOUND Laboratory, Cambridge Hearing Group, Clinical Neurosciences, University of Cambridge, Cambridge, United Kingdom; ^2^Audio Experience Design, Dyson School of Design Engineering, Imperial College London, London, United Kingdom; ^3^Wolfson College, Cambridge, United Kingdom

**Keywords:** spatial hearing, bilateral cochlear implants, binaural performance, dual task, remote testing, speech in noise, functional testing

## Abstract

Spatial hearing is critical for communication in everyday sound-rich environments. It is important to gain an understanding of how well users of bilateral hearing devices function in these conditions. The purpose of this work was to evaluate a Virtual Acoustics (VA) version of the Spatial Speech in Noise (SSiN) test, the SSiN-VA. This implementation uses relatively inexpensive equipment and can be performed outside the clinic, allowing for regular monitoring of spatial-hearing performance. The SSiN-VA simultaneously assesses speech discrimination and relative localization with changing source locations in the presence of noise. The use of simultaneous tasks increases the cognitive load to better represent the difficulties faced by listeners in noisy real-world environments. Current clinical assessments may require costly equipment which has a large footprint. Consequently, spatial-hearing assessments may not be conducted at all. Additionally, as patients take greater control of their healthcare outcomes and a greater number of clinical appointments are conducted remotely, outcome measures that allow patients to carry out assessments at home are becoming more relevant. The SSiN-VA was implemented using the 3D Tune-In Toolkit, simulating seven loudspeaker locations spaced at 30° intervals with azimuths between −90° and +90°, and rendered for headphone playback using the binaural spatialization technique. Twelve normal-hearing participants were assessed to evaluate if SSiN-VA produced patterns of responses for relative localization and speech discrimination as a function of azimuth similar to those previously obtained using loudspeaker arrays. Additionally, the effect of the signal-to-noise ratio (SNR), the direction of the shift from target to reference, and the target phonetic contrast on performance were investigated. SSiN-VA led to similar patterns of performance as a function of spatial location compared to loudspeaker setups for both relative localization and speech discrimination. Performance for relative localization was significantly better at the highest SNR than at the lowest SNR tested, and a target shift to the right was associated with an increased likelihood of a correct response. For word discrimination, there was an interaction between SNR and word group. Overall, these outcomes support the use of virtual audio for speech discrimination and relative localization testing in noise.

## Introduction

Speech testing plays a crucial role in the assessment of hearing function, including the evaluation of outcomes with hearing devices such as hearing aids or cochlear implants. There are a variety of speech testing materials, ranging from closed-set words to open-set sentence formats presented in quiet or in noise. A limitation of many of the current speech tests is that the listening skills assessed by the task are often different from those required for everyday communication environments. For instance, many tests were designed with a fixed speech source location and a co-located masker. This is the case for the Automated McCormick Toy Test (Summerfield et al., 1994), the Speech Reception in Noise Test [SPRINT (Cord et al., 1992; Brungart et al., 2017)], the Words in Noise Test [WIN (Wilson et al., 2007)], the Quick Speech in Noise Test (Killion et al., 2004), the Bamford-Kowal-Bench Speech in Noise Test [BKB-SIN (Etymotic Research Inc., 2005)], and the AzBio sentences lists (Spahr et al., 2012), among others. Conversely, in everyday environments, social interaction typically involves several talkers and sources of noise scattered around the listener. For communication to be successful, the listener needs to identify where the talker of interest is located and switch their focus rapidly as conversation unfolds.

It has been shown that tests using multi-talker babble or a single competing talker are sensitive to hearing status (Phatak et al., 2019), particularly if they target the use of “dip listening” – the ability to detect a signal in a fluctuating masker, which depends on accurate encoding of temporal fine structure information (Moore, 2014) – or quantify spatial release from masking (SRM), – the improvement in the detection of a signal in background noise arising from the spatial separation of the target signal and the background (Bronkhorst, 2000; Litovsky, 2012). Although tests of SRM can provide important diagnostic information about spatial hearing, they require that a speech-identification task is performed repeatedly as the location of the speech or noise is varied (Litovsky, 2012; Bizley et al., 2015), which makes them time-consuming. Moreover, unlike in real communication environments, the speech sources used in SRM testing have a fixed location at either the front or the sides of the listener.

In light of these limitations, Bizley et al. (2015) developed the Spatial Speech in Noise (SSiN) test as a tool for simultaneously evaluating SRM, localization, and speech discrimination performance in a background of multi-talker babble noise. The SSiN uses speech signals appropriate for adults and children, targeting discrimination of specific phonetic contrasts: complex vowel, simple vowel, initial consonant, and final consonant. These contrasts are represented by groups of four words each, so that testing is done in a closed-set discrimination paradigm. For example, for complex vowel, the four words within the group are “pale,” “peel,” “pile,” and “pool” (Table 1). The test features a speech-discrimination task in which the listener needs to report back two words within the group, the *reference* word and the *target* word, which are presented in succession. Simultaneously, listeners engage in a relative-localization task requiring that they report whether the target word was presented from the right or from the left of the location of the reference word. This dual-task approach and the use of multi-talker babble as a background noise were chosen to represent the challenges of listening in a complex communication environment. Unlike in typical SRM test setups, the locations of the sources of noise can be varied within the test session or across versions, as will be described later, and the speech-source locations change from trial to trial. Further work was carried out by Ahnood (2017) and Parmar et al. (2018) in order to adapt the SSiN for use with people with hearing aids and cochlear implants. Modifications included increasing the spacing between loudspeakers and restricting the number of noise sources during the task in order to make the task feasible to listeners with hearing loss.

**TABLE 1:** Word groups by target phonetic contrast and word items within each group.

Target phonetic contrast	Word items			
Complex vowel (Vc)	Pale	Pool	Pile	Peel
Simple vowel (Vs)	Hoot	Heat	Heart	Hurt
Initial consonant (Ci)	Chalk	Talk	Fork	Stork
Final consonant (Cf)	Cheat	Cheese	Cheap	Cheek

While the SSiN is an efficient way to simultaneously assess speech discrimination and relative localization, a key element of its setup is the use of a loudspeaker array simulating a AB-York Crescent of Sound (Kitterick et al., 2011) to deliver the stimuli. Implementations based on loudspeaker arrays are costly both in terms of material and spatial requirements. Additionally, this test require a face-to-face visit. The constraints imposed by the current COVID-19 pandemic have accelerated the development and adoption of tele-audiology practices (Ayas et al., 2020; Saunders and Roughley, 2020; Parmar et al., 2021). Remote-health applications that enable users to complete diagnostic tests and submit them to their clinical departments are very much in demand.

One solution to the spatial and economic costs of multi-loudspeaker arrays, and a response to the demand for remote clinical testing, is to use binaural spatialization to render complex listening environments which can be delivered to the listener using a pair of headphones (Cuevas-Rodríguez et al., 2019). The most common implementation of the binaural spatialization technique is based on the Head-Related Transfer Function (HRTF), which embeds localization cues – such as Interaural Time Differences (ITDs), Interaural Level Differences (ILDs), and spectral cues – within the original sound stimuli (Blauert, 1997). The capabilities of binaural spatialization for generating complex soundscapes are virtually unlimited in terms of the number and location of sound sources and their relative distance, as well as the characteristics of the simulated room (e.g., large halls, small studios, etc.) (Cuevas-Rodríguez et al., 2019). Additionally, the requirements for playback devices are simple. It is possible to use a standard pair of headphones connected to the computer audio output, or wireless streaming for hearing devices, for the delivery of the sounds. For these reasons, binaural spatialization could have a major impact when applied to audiological testing (Pausch and Fels, 2019; Keidser et al., 2020).

In spite of their great potential, there are only a few examples of clinical-audiology applications that use binaural spatialization. For instance, the Listening in Spatialized Noise Sentences test [LiSN (Cameron and Dillon, 2008)] assesses stream segregation by adaptively estimating the speech reception threshold (SRT) for sentences in a competing background. Pitch cues (identity of the target talker vs. identity of the talker/s in the background) and/or spatial cues (co-located or ±90° azimuth separation) are varied during the test. Another example of a virtual-audio clinical-audiology application is the Auditory Speech Sound Evaluation (A§E^®^, © P.J. Govaerts, Antwerp, Belgium) ILD Sound Localization Test, which uses two loudspeakers to simulate thirteen spatial locations by introducing ILDs on a 4000-Hz narrow band of noise (Otoconsult Helpdesk, 2021).

The SSiN has some advantages over these examples. First, it uses smaller intervals than the LiSN for spatial-discrimination testing. Second, it uses more meaningful stimuli than the A§E ILD sound localization test, with a wider frequency range. However, it still has the limitation of requiring a complex set-up. A virtual-audio version of the SSiN, the SSiN-VA, was implemented. SSiN-VA retains the SSiN capabilities for testing speech discrimination and relative localization while minimizing any space and equipment requirements. The aim of this project was to determine whether the patterns of responses as a function of spatial location obtained with the SSiN-VA are similar to those previously obtained with the SSiN test for normal-hearing listeners. Our hypothesis was that the SSiN-VA leads to patterns of word discrimination and relative localization similar to those previously obtained with the SSiN. We investigated this hypothesis by conducting the SSiN-VA with 12 normal-hearing participants. Our predictions, based on existing SSiN data, were that, for relative localization, performance would deteriorate at the lateral locations relative to performance at the midline and that, for word discrimination, performance would be best at the lateral locations and lowest around the midline. Additionally, we hypothesized that performance for both word discrimination and relative localization increases the higher the signal-to-noise ratio (SNR) at which the test was conducted. This is a novel aspect of this work, as the influence of SNR on performance has not been assessed for the SSiN. Further, assessing the effect of SNR on SSiN-VA outcomes was of interest as there was no knowledge of the difficulty of the task for the participants, given the virtual setup. Lastly, we hypothesized that:

For relative localization:

a) Performance is similar across word groups as their overall audibility is equivalent.
b) The effect of SNR is similar across spatial locations of the speech sources (no interaction between SNR and the spatial location of the speech).
c) There is no direction bias in the responses of the participants. In other words, a correct response is equally likely for trials where the target shifts to the left and trials where the target shifts to the right. If a direction bias were found, it would be investigated whether the bias is present regardless of the location of the speech sources (no interaction between spatial location and direction of the shift) and for all SNRs (no interaction between SNR and direction of the shift).

For word discrimination:

a) Word discrimination varies across word groups as the spectral cues required for correct discrimination of each group may differ in their vulnerability to being masked by the babble noise.
b) The order of the words (i.e., whether a word is target or reference) does not influence performance.
c) Changing the location of the speech source (azimuth) might lead to changes in the SNR at each ear, and this affects performance for different word groups unevenly (interaction between word group and azimuth).
d) Increasing the SNR improves word discrimination regardless of azimuth (no significant interaction between SNR and azimuth).
e) The effect of SNR is stronger for word groups where the speech sounds key to the phonetic contrast is lowest in level, such as the initial-consonant and the final-consonant groups (interaction between SNR and word group).

Finally, the patterns of responses for SSiN-VA were graphically compared to those obtained with a dataset obtained with a loudspeaker spatial setup similar to the one simulated here.

## Materials and Methods

### Participants

Twelve participants (eight female, four male) with normal hearing were tested. Their median age was 26 years, ranging from 21 to 52 years (mean 28.58, SD = 8.73). All participants had air-conduction hearing thresholds for octave frequencies in the range 250–8000 Hz equal to or better than 20 dB HL, or a maximum of one frequency with threshold equal to 25 dB HL, as measured with an Interacoustics Affinity audiometer in a quiet room.

The experiment designs for preliminary work were reviewed and approved by the Joint Research Compliance Office at Imperial College (Ref. 19IC5073). The main experiments were reviewed and approved by the Cambridge Psychology Research Ethics Committee (Ref. 2019.093).

### Implementation of the Spatial Speech in Noise-Virtual Acoustics Test

As mentioned, the test used here, the SSiN-VA, was an adaptation of the SSiN Test developed by Bizley et al. (2015). The basic structure of the SSiN-VA was the same as that for the SSiN: In each trial, a reference word was presented from one of the loudspeaker locations. The reference word was followed by a target word, which was presented from an adjacent loudspeaker location. Simultaneously, sixteen-male-talker babble (Huckvale, 1989) was presented to the listener. The listener was required to provide a speech-discrimination response by selecting the reference word and the target word from four buttons, each corresponding to one word. Additionally, the listener provided a relative-localization response by indicating in which direction the location of the target word shifted relative to the reference word. This was done by using one of two buttons labeled “left” and “right,” respectively. The test used speech material taken from a closed-set children’s speech discrimination test, the Chear Auditory Perception Test [CAPT (Marriage et al., 2011; Vickers et al., 2018)]. Each word belonged to one of four closed-set groups. Each group contained four words which possessed a particular type of phonetic contrast; the words differed in a complex vowel (pale, pool, pile, or peel), a simple vowel (hoot, heat, heart, or hurt), initial consonant (chalk, talk, fork, or stork), or a final consonant (cheat, cheese, cheap, or cheek), as explained above and shown in Table 1.

**FIGURE 1:**
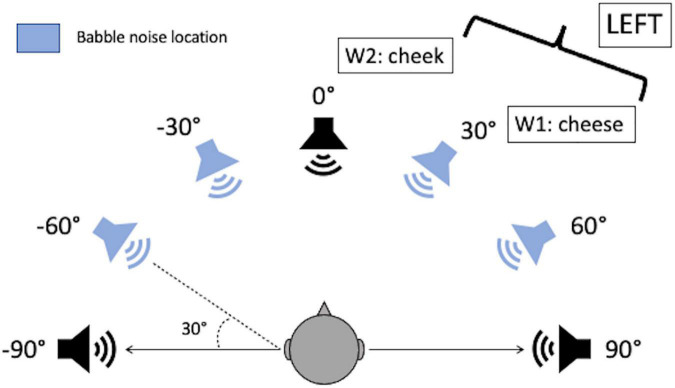
Diagram of the simulated loudspeaker locations around the listener showing seven sound sources separated at 30° azimuth intervals. An example of a trial is given where the sources of the reference word (W1) and the target word (W2) are represented on the diagram, and the correct relative-localization answer is given. The diagram shown to the participant was identical except that no indication of the spatial location of the babble or the azimuth were given.

There are some differences between the original SSiN test and the SSiN-VA other than the use of headphones instead of loudspeakers. These changes were introduced in order to make the test more feasible for users with hearing loss. For the SSiN-VA, the number of spatial locations was reduced from from 13 to seven, and the spacing between the sources used in a given trial was doubled compared to the first implementation of the SSiN. Thus, the SSiN-VA used azimuths corresponding to −90°, −60°, −30°, 0°, 60°, 30°, and 90° (Figure 1). Intervals of 15°, such as those used in the SSiN, may be too small for people with hearing loss to be able to perform the relative-localization task above chance (Ahnood, 2017; Parmar et al., 2018). In addition, for the SSiN-VA, the babble was constantly delivered from four spatial locations: −60°, −30°, 60°, and 30°, instead of simultaneously from all loudspeaker locations as in Bizley et al. (2015). Delivering the babble from all loudspeaker locations may make the test too challenging for people with hearing loss (Ahnood, 2017; Parmar et al., 2018). This prompted other researchers to reduce the number of babble source for this task. For instance, Ahnood (2017) delivered the noise from either the −60° and −30° locations, or the 30° and 60° locations (i.e., from two sources at a time, alternative from the right or the left hemispace). Note that these are the same locations that are used here, but with only two sources within the same hemispace active at a given trial. Although this maximizes the amount of SRM that can be obtained by increasing the distance between the sources of the speech and the sources of the babble for some of the trials, the number of trials needs to be large enough to be able to accurately represent both noise-location configurations. Because the ultimate objective of the SSiN-VA is for it to be used clinically, it was decided that the noise would be consistently delivered from the reduced set of locations used by Ahnood (2017) but in a simultaneous manner from all four sources. This made it possible to reduce the number of trials collected and simplify the study design.

The SSiN-VA prototype was created using MaxMSP (Cycling’74, 2021) and the 3D Tune-In Toolkit (Cuevas-Rodríguez et al., 2019), specifically its Virtual Studio Technology (VST) implementation (Picinali et al., 2019a). One instance of the VST plugin, loaded with a KEMAR mannequin HRTF from the SADIE II database (Armstrong et al., 2018), was used to spatialize each individual virtual loudspeaker. No room-acoustics simulation was performed, therefore the spatialization was fully anechoic. The ITDs were individualized for each participant by measuring their head circumference and inputting it in the 3D Tune-In Toolkit rendering engine. More details about how the spatialization was performed accounting for this measure can be found in Cuevas-Rodríguez et al. (2019). A head tracker was used to update the locations of the virtual loudspeakers every 12 ms in order to ensure that the rendered virtual sound field was anchored to the surrounding space rather than rotating with the head of the listener, as it happens in real environments when listening to audio reproduced from an array of loudspeakers. More details about the HRTF interpolation processes implemented in order to simulate the movement of the head relative to the virtual loudspeakers can be found in Cuevas-Rodríguez et al. (2019). The importance of accounting for head movements when reproducing binaural signals is well documented in the literature (Begault et al., 2001). Even though participants were instructed to look at the front, it was decided to implement head tracking in order to make the experience as close as possible to the original SSiN test, as it is known that small head movements can have a dramatic impact on SRM, significantly improving performance (Grange and Culling, 2016). The timing of the sequence playback was arranged as follows: babble onset at 0 s, first word at 0.5 s, second word at 2 s, and babble offset at 3.5 s. Each word was approximately 1 s long.

### Equipment for the Spatial Speech in Noise-Virtual Acoustics Test

Stimuli were presented using a MacBook Pro via Sennheiser HD-600 headphones. An Apple iPhone 5 was used as head tracker, and was mounted on the top of the headphones. The app GyrOSC was used to send the head-tracking data through WiFi to the MacBook Pro via Open Sound Control (OSC, Freed, 1997).

### Procedure for the Spatial Speech in Noise-Virtual Acoustics Test

#### Calibration and Presentation Level

Scaling of the word stimuli was performed by calculating the root-mean-square (RMS) levels of the steady-state portions of the vowels within the words as identified independently by two researchers using Praat software, version 6.1.14 (Boersma and Weenink, 1992). Where discrepancies occurred, a third researcher was involved in discussion. A MATLAB (The MathWorks Inc., 2019) script was used to adjust the RMS levels of the word stimuli so that the levels of the steady-state portions of the vowels were equal across words. Appropriate scaling of the background noise was performed taking into account the number of sources in order to achieve the same RMS level as for the word stimuli. Presentation levels were calibrated using a Tektronix MDO3024 Mixed Domain Oscilloscope using the headphone sensitivity data to calculate the voltage required to deliver the sound level required for the calibration noise. Because the desired playback level for the word stimuli was 52 dB SPL when the SNR was specified as 0, the RMS level of the calibration noise was set 20 dB above the RMS level of the word stimuli, and the calibration noise was played back at 72 dB SPL. The level of the multi-talker babble was kept at 52 dB SPL throughout the task (consistent with Bizley et al.’s implementation). The level of the speech was varied across runs in order to collect data for three different SNRs as described below.

#### Speech Reception Threshold Determination

First, the speech reception threshold (SRT) for each participant was determined by presenting words from a simulated azimuth of 0° (i.e., from the frontal location) while the multi-talker babble was simultaneously delivered from −60°, −30°, 60°, and 30°. In each trial, one word, randomly selected from the sixteen used in the test, was presented. Participants were shown the discrimination group that contained the correct word. For example, if the word presented was “peel,” participants were shown the words “pool,” “pile,” “pale,” and “peel.” Participants were required to click on the word that they heard. The SNR was 0 dB in the first trial (calibration details given in section “Calibration and Presentation Level”), and was adaptively varied in 2-dB steps following a one-up one-down technique in subsequent trials. The test stopped after eight reversals were obtained. The SRT was calculated as the average SNR at the last six reversals.

Once the SRT was measured, the SNRs for three conditions were calculated: (1) the individually measured speech recognition threshold (SRT), determined as explained above; (2) SRT + 3 dB; (3) SRT + 6 dB. This was to address the aim of determining whether there was an effect of SNR on performance for both relative localization and word discrimination.

#### The Spatial Speech in Noise-Virtual Acoustics Task

**FIGURE 2:**
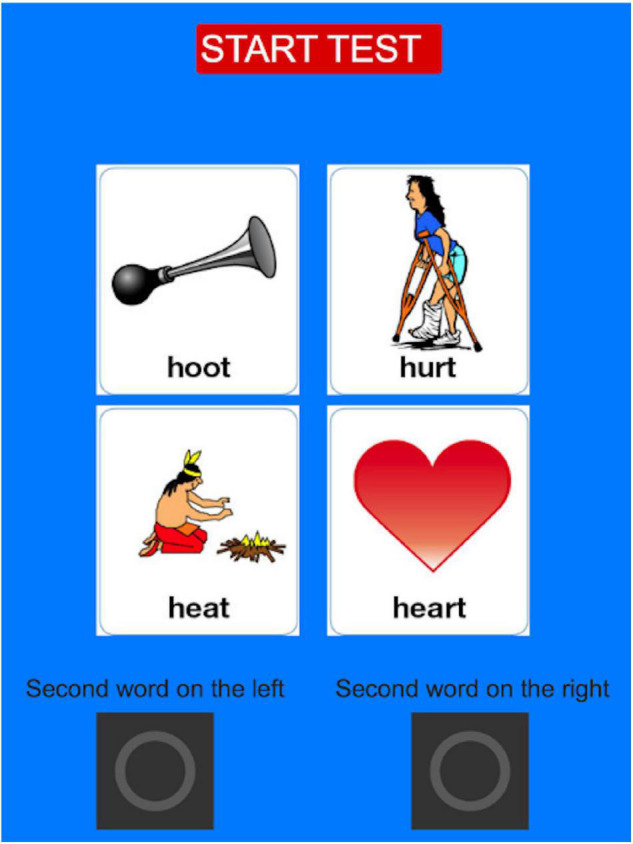
Test interface. Participants clicked on each of two words presented in each trial and on the “**right**” or “**left**” buttons to indicate the location of the target word relative to the reference word.

Before testing started, participants were introduced to the task by being shown a diagram of the simulated loudspeakers around the listener. They were told that they might have the impression that words came from the locations shown in the diagram, and that in each trial two words would be presented. The second word would come from either the right or the left with respect to the first word. Their task was to report the two words that they heard, in order, and the location of the second word with respect to the first one. Examples were given using cards with words written on them, as shown in Figure 1, which were moved around the diagram to simulate possible trials and verify that the participant had understood the task. Next, approximately six trials of the task were presented to the participant at SNR = 0. Participants used the interface shown in Figure 2 to provide their answers. After this, the participant was asked whether they had understood the task. If they confirmed that they had, testing began.

The three test conditions were administered in three blocks of two runs each. Each run took approximately 12–15 min. Blocks were presented in a pseudorandom order, using all possible orders across the twelve participants who took part. Each run was tested using a 96-trial list. Each trial was characterized by a reference word, a target word, and a simulated location for each of them. There were eight trials for each reference-target location pair. Each of the four word-discrimination groups was played twice from each reference-target location pair. Responses where the participant pressed buttons repeatedly or pressed extra buttons led to inaccurate logging. These trials were discarded prior to analysis. One random additional trial was presented in each run due to an implementation flaw. The responses to these trials were not discarded as the additional run was not associated with a particular condition. The total number of trials presented to each participant was 582 (97 trials * 6 runs). After discarding spurious trials, participants contributed an average of 577 trials each to the analysis phase (range 561–582).

### Analyses

#### Statistical Analysis

Statistical analysis was based on trial-by-trial responses. The response variables were binary, with two possible outcomes for “correct response”: yes or no. The *glmer* function within the *lme4* package version 1.127 (Bates et al., 2015), in R version 4.1.0 (R Core Team, 2021), was used to fit a mixed-effects maximum likelihood binary logistic multilevel model separately for relative localization and word discrimination. The aim of the analysis was to determine whether the patterns of word discrimination and relative localization were similar to those previously obtained with the SSiN test. If this were the case, the outcomes of the analysis would be that performance for relative localization and speech discrimination is predicted by the spatial location of the speech sources. For relative localization it was expected that the likelihood of a correct response grew with increasing proximity of the speech sources to the midline. For speech discrimination, the opposite pattern was expected, i.e., that the likelihood of a correct response decreased with increasing proximity of the speech sources to the midline. The effect of word group and SNR as predictor for each task and the effect of the direction of the shift from reference to target in the relative-localization task was also assessed. In addition, some interactions between the predictors were investigated, as described below.

For relative localization, the “mean location” was defined as the average location of the pair of spatial locations of the target and the reference word within each trial, following Bizley et al. (2015), Ahnood (2017), and Parmar et al. (2018). The rationale for this is that, for a given pair of simulated loudspeakers, the participants would have had to make a localization judgment based on binaural localization cues of equal magnitude, albeit with opposite directions (Bizley et al., 2015). The model included a random intercept by participants in order to control for the non-independence of the data (Winter, 2019). The model was progressively built up by successively including the following predictors as fixed effects: “mean location” (−75°, −45°, −15°, 15°, 45°, or 75°), SNR (0, 3, or 6 dB above the SRT), direction of the shift from the target to the reference word (right or left), and word group (simple vowel, complex vowel, initial consonant, or final consonant). The following interactions were investigated: SNR × Mean Location, Direction × SNR, Direction × Mean Location.

For word discrimination, the model had a random intercept by participants to account for the fact that the participants were repeatedly tested. Because some participants were well above the 50%-word discrimination mark for the easiest condition (due to overestimation of the SRT), it was assumed that the slopes for SNR would vary across participants. Thus, a random slope for SNR was included. Next, fixed effects for SNR, azimuth, word order (i.e., whether the word was the target of the reference word), and word group were introduced one by one to build up the model. Interaction terms were included for word group × SNR, word group and azimuth, and azimuth and SNR.

The predictors of each model had their variance inflation factors (VIFs) calculated to ensure that multi-collinearity was not present. Models were compared by performing likelihood ratio tests. If any two models compared were not statistically different, the less complex model was chosen. *Post hoc* pairwise comparisons were carried out using the *multcomp* package (Hothorn et al., 2008) and Bonferroni corrections were applied.

#### Comparison With Data Previously Collected With Loudspeakers

Outcomes were plotted and compared with data previously collected by Ahnood (2017) while completing an MSc dissertation at University College London, supervised by Jennifer Bizley and author Deborah Vickers. This dataset was chosen because it was obtained using the same number and distribution of azimuths as in the present work in a similar population. Ahnood (2017) tested 12 normal-hearing adults using an implementation of the SSiN test which delivered the background babble alternatively from two loudspeakers placed *either* at −60° and −30° azimuth *or* at 30° and 60° azimuth. In other words, in each trial, the background babble came either from the left or from the right of the listeners. Conversely, in our implementation, the background babble was symmetrically delivered from these same four loudspeaker locations in all trials. In spite of the differences in the location of the noise sources relative to the speech sources, a comparison across these datasets can be insightful as to whether the SSiN-VA leads to similar patterns of spatial hearing compared with the SSiN. It is expected that the shape of the performance-by-location function is more similar across datasets for the relative localization data. The speech discrimination outcomes are likely to be more strongly influenced by the spatial separation between the speech sources and the noise sources with respect to the listener’s ears.

## Results

**FIGURE 3:**
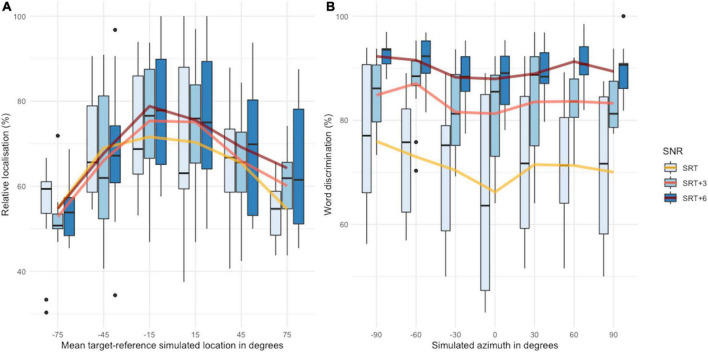
SSiN-VA performance for relative localization **(A)** and word discrimination **(B)**. Boxplot colors indicate the SNR at which the test was done. The black lines within the boxplots represent the median. Mean scores are joined with a thick line across spatial locations, separately for each SNR. Figure made using the packages ggplot2 (Wickham, 2016) and cowplot (Wilke, 2020) in R (R Core Team, 2021).

Figure 3 shows relative-localization and word-discrimination performance for each of the SNRs at which the participants were tested. Relative localization performance is plotted as a function of the mean target-reference location. For each of the SNRs tested, the function has the shape of an inverted *U*. This means that performance tended to be better at the midline than at the lateral locations. For word discrimination, performance tended to be slightly better at the lateral locations than at the midline (i.e., followed a *U*-shaped pattern), although this trend was more evident for the responses obtained with SNR = SRT. The effect of SNR can also be seen in this figure. For relative localization, responses varied somewhat with SNR; but for speech discrimination, the effect of SNR led to large improvements in performance. Statistical analyses were conducted to determine whether these trends were significant in order to test the hypotheses stated in the introduction.

### Statistical Analysis

#### Relative Localization

For relative-localization performance, it was hypothesized that performance would resemble the pattern typically obtained with loudspeakers, with relative localization being better near the midline than at the lateral locations. Thus, the mean location of the target and reference words was expected to have an impact on performance. This was confirmed by comparing a random intercept only model with one where mean location was added as a fixed effect [*c*^2^(5) = 177.86, *p* < 0.001]. SNR was hypothesized to affect performance. This was confirmed by adding SNR as a fixed effect, which significantly improved the model’s fit [*c*^2^(2) = 9.42, *p* = 0.0090]. The direction of the spatial shift from the reference word to the target word was thought not to have an impact on performance. However, its addition as a fixed effect significantly improved the model’s fit [*c*^2^(1) = 23.85, *p* < 0.001], indicating a direction bias. Word group was not expected to influence performance, which was confirmed [*c*^2^(3) = 6.76, *p* = 0.0798]. The impact of SNR did not vary across mean locations [*c*^2^(10) = 10.33, *p* = 0.4123], and the direction bias effect did not vary across SNRs [*c*^2^(2) = 1.64, *p* = 0.4414] or mean locations [*c*^2^(5) = 10.08, *p* = 0.0730]. In summary, relative localization performance was predicted by the mean location of the source and the target word, the SNR, and the direction of the spatial shift from the reference to the target word. Performance did not significantly differ across word groups. No significant interactions were found between SNR and mean locations, and between direction and SNR or mean location.

Table 2 summarizes the odds ratios, 95% confidence intervals (CIs), and *p*-values of the final model. As the variables were treatment-coded, the intercept represents the likelihood of a correct response when all variables are set to 0: mean location −75°, SNR = SRT, direction left, and word group = 1, complex vowels. The intercept was −0.04 (SE = 0.16, *p* = 0.7984, odds ratio = 0.96). This indicates that, for this condition, the likelihood of a correct response was only slightly lower than the likelihood of an incorrect response. All other estimates of the model are referenced to this condition.

**TABLE 2:** Outcomes of the statistical analysis for SSiN-VA relative-localization performance.

	Relative Localization Performance		
*Predictors*	*Odds Ratios*	*CI*	*p*
(Intercept)	0.96	0.71–1.31	0.798
Mean Location −75°	*Reference*		
Mean Location −45°	1.82	1.53–2.17	**<0.001**
Mean Location −15°	2.71	2.26–3.24	**<0.001**
Mean Location 15°	2.49	2.08–2.98	**<0.001**
Mean Location 45°	1.76	1.48–2.09	**<0.001**
Mean Location 75°	1.27	1.07–1.51	**0.006**
SNR SRT	*Reference*		
SNR SRT + 3 dB	1.08	0.95–1.22	0.250
SNR SRT + 6 dB	1.22	1.07–1.38	**0.002**
Direction left	*Reference*		
Direction right	1.29	1.17–1.43	**<0.001**
**Random Effects**			
σ^2^	3.29		
τ_00 Participant_	0.23		
ICC	0.06		
N _Participant_	12		
Observations	6924		
Marginal R^2^/Conditional R^2^	0.040/0.102		

*Table generated using the package sjPlot (Lüdecke, 2021). Bold values correspond to statistically significant outcomes.*

**FIGURE 4:**
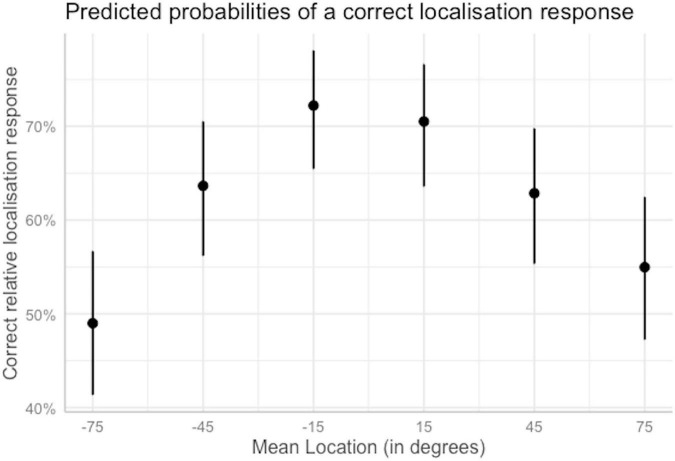
Predicted probabilities of a correct localization response as a function of the mean location of the target and reference words when other predictors are held at their reference level. Plot obtained using the sjPlot package (Lüdecke, 2021). Error bars indicate 95% confidence intervals.

Assessing how the odds of a correct response varied when shifting mean location from left to right is helpful to characterize the shape of the performance function. This made it possible to test the hypothesis that performance would be better at the midline than at the lateral locations. When the mean location of the target and reference was −45° or 45°, the odds of a correct response significantly increased by 1.82 and 1.76 times, respectively, compared to −75°. This means that for mean locations −45° and 45°, a correct response was 1.75 and 1.70 times more likely than an incorrect response, respectively. Greater increases, by 2.71 and 2.49 times with respect to the intercept, respectively, were found when mean location −75° was compared with −15° and 15°. This suggests that for mean locations −15° and 15°, a correct response was 2.60 and 2.39 times more likely than an incorrect response. Finally, and against expectations, when the mean location of the target and reference was 75°, the odds of a correct response increased significantly, by 1.27 times, with respect to −75°. Thus, a correct response for mean location 75° was 1.23 times more likely than an incorrect response. In spite of this asymmetry, these outcomes are overall consistent with the expected inverted-*U* shape of relative localization as a function of mean location. The odds reported here were transformed into percentages and are illustrated in Figure 4.

**FIGURE 5:**
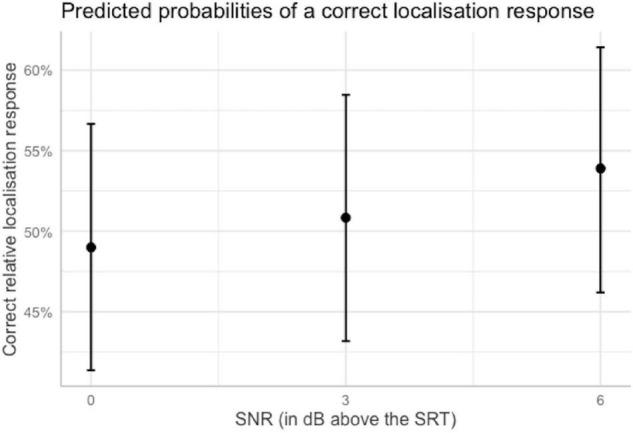
Predicted probabilities of a correct localization response as a function of the SNR at which the stimuli were presented when other predictors were held constant at their reference level. Plot obtained using the sjPlot package (Lüdecke, 2021). Error bars indicate 95% confidence intervals.

Comparing the odds of a correct response across SNRs is helpful to evaluate the hypothesis that performance increases with increasing SNR. The odds of a correct response for relative localization significantly increased by 1.22 times when the SNR was raised by 6 dB with respect to the SRT. This means that at SNR = SRT + 6 dB, a correct response was 1.17 times more likely than an incorrect response, compared to 0.96 times for SNR = SRT. Although this increase is small, it confirms one of our hypotheses that performance would improve with increasing SNR. An increase of the SNR by 3 dB above the SRT failed to significantly increase the odds of a correct response. The odds reported here were transformed into percentages and are illustrated in Figure 5.

**FIGURE 6:**
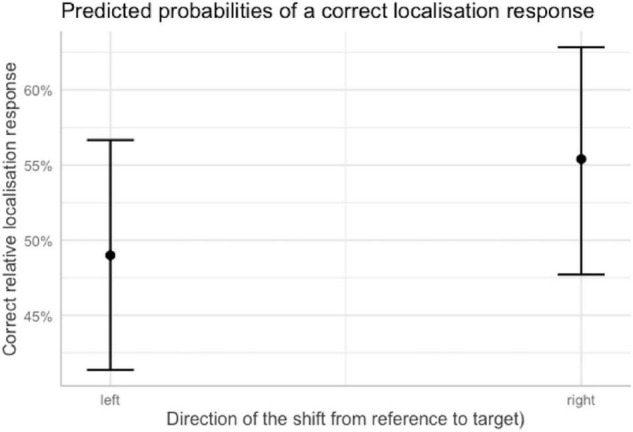
Predicted probabilities of a correct localization response as a function of the direction of the shift from reference to target word, when other predictors were held constant at their reference level. Plot obtained using the sjPlot package (Lüdecke, 2021). Error bars indicate 95% confidence intervals.

Comparing the odds of a correct response for each direction of the spatial shift from reference to target suggested that when the shift was toward the right, participants were more likely to obtain a correct answer. The odds of a correct response increased by 1.29 times with respect to a shift toward the left, making a correct response 1.24 times more likely than an incorrect one when the shift from reference to target was toward the right. This indicates a bias in this direction, contrary to what was hypothesized in the introduction, i.e., that there would not be a direction bias. Figure 6 shows the reported odds transformed into percentages.

*Post hoc* analysis was performed in order to compare average performance across pairs of mean locations, SNRs, word groups, and shift directions, using the *ghlt* function within the *multcomp* package (Hothorn et al., 2008). Bonferroni corrections were applied to account for repeated testing. Outcomes are shown in Table 3. These comparisons revealed that performance at the most eccentric mid locations (−75° and 75°) was significantly lower than performance at the most central mid locations of −15° and 15°, which in turn were not significantly different from each other. Additionally, performance at −45° and 45° was significantly lower than performance at −15°, but not significantly different from performance at 15°. Performance at −75° was significantly lower than performance at 45° but not significantly lower than performance at −45°. Again, the pattern was consistent with an inverted-*U* shape, although, statistically, there were some asymmetries in the data. For SNR, *post hoc* pairwise comparisons indicated that, across mean locations, word groups, and shift directions, performance was significantly lower for SNR = SRT than for SNR = SRT + 6. For the direction of the shift, the comparison between left and right continued to be significant.

**TABLE 3:** *Post hoc* analysis for relative localization.

Comparison	Estimate	Std. Error	*z*-value	Pr(> | z|)
**Mean Location**				
−75° vs. −45°	−0.60	0.09	−6.77	**<0.001**
−75° vs. −15°	−1	0.09	−10.76	**<0.001**
−75° vs. 15°	−0.91	0.09	−9.96	**<0.001**
−75° vs. 45°	−0.57	0.09	−6.41	**<0.001**
−75° vs. 75°	−0.24	0.09	−2.77	0.083
−45° vs. −15°	−0.39	0.09	−4.16	**<0.001**
−45° vs. 15°	−0.31	0.09	−3.31	**0.014**
−45° vs. 45°	0.03	0.09	0.38	1
−45° vs. 75°	0.36	0.09	4.04	**<0.001**
−15° vs. 15°	0.08	0.10	0.86	1
−15° vs. 45°	0.43	0.09	4.54	**<0.001**
−15° vs. 75°	0.76	0.09	8.13	**<0.001**
15° vs. 45°	0.35	0.09	3.69	**0.003**
15° vs. 75°	0.67	0.09	7.31	**<0.001**
45° vs. 75°	0.3	0.09	3.68	**0.003**
**SNR**				
SRT vs. SRT + 3 dB	−0.7	0.06	−1.15	0.749
SRT vs. SRT + 6 dB	−0.20	0.06	−3.04	**0.007**
SRT + 3 dB vs. SRT + 6 dB	−0.12	0.06	−1.89	0.177
**Direction**				
left vs. right	−0.26	0.05	−4.87	**<0.001**

*P-values were Bonferroni corrected. Bold values correspond to statistically significant outcomes.*

#### Word Discrimination

For word discrimination, it was hypothesized that performance would resemble the pattern typically obtained with loudspeakers, with word discrimination being worse near the midline than at the lateral locations. Thus, the spatial location of the source (azimuth) was expected to be influential. This was confirmed, as a random-intercept-random-slope only model (random intercept by participant and random slope by SNR) was significantly worse than an identical model which had azimuth as a fixed effect to predict the discrimination outcome [*c*^2^(6) = 35.42, *p* < 0.001]. Performing the task at different SNRs was thought to influence performance, which was the case [inclusion of SNR as fixed effect, *c*^2^(2) = 26.20, *p* < 0.001]. As expected, different word groups led to varying levels of word discrimination performance [word group, *c*^2^(3) = 962.03, *p* < 0.001]. To assess whether the order of presentation of the words within each trial (i.e., whether the word was target or reference) was associated with an increased likelihood of a correct response, word order was included. However, this failed to improve the predictions of the model [*c*^2^(1) = 1.51, *p* = 0.2192]. Increasing SNR should lead to better word discrimination independent of azimuth, which was the case [no significant interaction between SNR and azimuth, *c*^2^(12) = 6.91, *p* = 0.8632]. As the different word groups might be more or less susceptible to masking by the level of the babble noise, the interaction term of SNR × word group was included, resulting in improved fit [*c*^2^(6) = 119.37, *p* < 0.001]. Presenting words from varying azimuths changes the SNR at each ear, thus possibly making some word groups easier to understand than others. However, inclusion of the interaction azimuth × word group significantly worsened the model’s fit [*c*^2^(6) = 119.37, *p* < 0.001]. In summary, performance for word discrimination was predicted by the location of the speech source (azimuth), and by SNR and word group. Additionally, there was an interaction between SNR and word group. Thus, the effects of these two factors need to be considered jointly, as will be done below. The effect of SNR or word group did not vary with azimuth. The order of presentation of the words within each trial did not influence performance.

**FIGURE 7:**
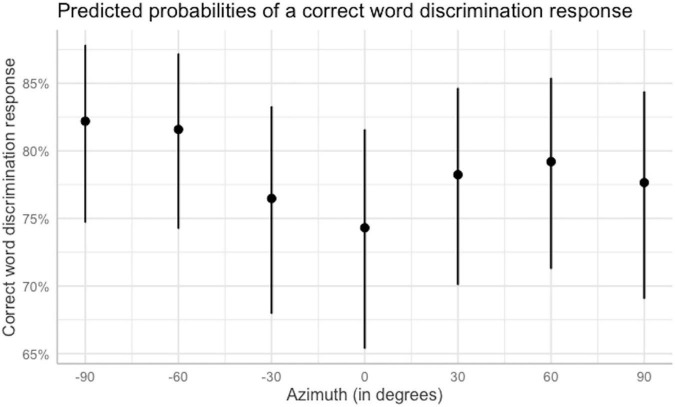
Predicted probabilities of a correct word-discrimination response as a function of azimuth when other predictors are held at their reference level. Error bars indicate 95% confidence intervals. Plot obtained using the sjPlot package (Lüdecke, 2021).

Table 4 reports the odds ratios, 95% CIs, and *p*-values for the final model. As the variables were treatment-coded, the intercept represents the likelihood of a correct response when all variables were set to the reference level for each one of them (azimuth = −90°, SNR = SRT, word group = 1, complex vowels). The intercept was 1.53 (SE = 0.23, *p* < 0.001, odds ratio = 4.62). Therefore, for this condition, a correct word identification response was 4.62 times more likely than an incorrect response. Again, assessing how the odds of a correct response vary as a function of azimuth is useful to characterize the shape of the performance function. Having the speech coming from −60° or 60° did not significantly affect the odds of a correct response compared to −90°. However, having the speech coming from −30° or 30° significantly decreased the odds of a correct response, compared to −90°, by 0.70 and 0.78 times, respectively, making a correct response 3.23 and 3.60 times more likely than an incorrect response, respectively. Further, when the speech came from 0°, the odds of a correct response decreased even further, by 0.63 times, making a correct response 2.91 times more likely than an incorrect one. Finally, contrary to prior expectations, the odds of a correct response were significantly lower for the rightmost location in space (azimuth = 90°) compared to the left-most location in space (azimuth = −90°) by 0.75 times, making a correct response 3.47 times more likely than an incorrect one. Overall, these outcomes were consistent with the expected *U*-shaped performance function, although displaying an asymmetry in performance between −90° and 90°. The reported odds were transformed into percentages and are illustrated in Figure 7.

**TABLE 4:** Outcomes of the statistical analysis for SSiN-VA word-discrimination performance.

	Word Discrimination Performance		
*Predictors*	*Odds Ratios*	*CI*	*p*
(Intercept)	4.62	2.95–7.22	**<0.001**
Azimuth −90°	*Reference*		
Azimuth −60°	0.96	0.78–1.18	0.701
Azimuth −30°	0.70	0.57–0.86	**0.001**
Azimuth 0°	0.63	0.51–0.77	**<0.001**
Azimuth 30°	0.78	0.63–0.96	**0.017**
Azimuth 60°	0.82	0.67–1.01	0.069
Azimuth 90°	0.75	0.60–0.95	**0.018**
SNR SRT	*Reference*		
SNR SRT + 3 dB	3.02	2.24–4.07	**<0.001**
SNR SRT + 6 dB	6.68	4.53–9.84	**<0.001**
Word Group V_c_	*Reference*		
Word Group V_s_	0.76	0.62–0.92	**0.005**
Word Group C_i_	1.22	1.00–1.50	0.056
Word Group C_f_	0.37	0.31–0.45	**<0.001**
SNR SRT + 3 dB: Word Group V_s_	0.96	0.69–1.35	0.832
SNR SRT + 3 dB: Word Group C_i_	0.89	0.63–1.27	0.522
SNR SRT + 3 dB: Word Group C_f_	0.46	0.34–0.63	**<0.001**
SNR SRT + 6 dB Word Group V_s_	1.08	0.70–1.67	0.712
SNR SRT + 6 dB Word Group C_i_	1.39	0.84–2.30	0.202
SNR SRT + 6 dB Word Group C_f_	0.28	0.20–0.41	**<0.001**
**Random Effects**			
σ^2^	3.29		
τ_00 Subject_	0.47		
τ_11 Subject.SNR3_	0.09		
τ_11 Subject.SNR6_	0.15		
ρ_01_	−0.65		
	−0.82		
ICC	0.09		
N _Subject_	12		
Observations	13848		
Marginal R^2^/Conditional R^2^	0.237/0.304		

*Table generated using the package sjPlot (Lüdecke, 2021). Bold values correspond to statistically significant outcomes.*

Increasing the SNR from SRT to SRT + 3 dB and from SRT to SRT + 6 dB led to a greater likelihood of a correct response, consistent with our hypothesis of better performance the higher the SNR. Changes in word group from complex vowels to simple vowels and from complex vowels to final consonant significantly decreased the likelihood of a correct response. However, it should be noted that, as treatment coding was used and a significant interaction between SNR and word group was found, it is not possible to assess the effects of SNR and word group separately. The interaction was explored using *post hoc* analysis as detailed below.

**FIGURE 8:**
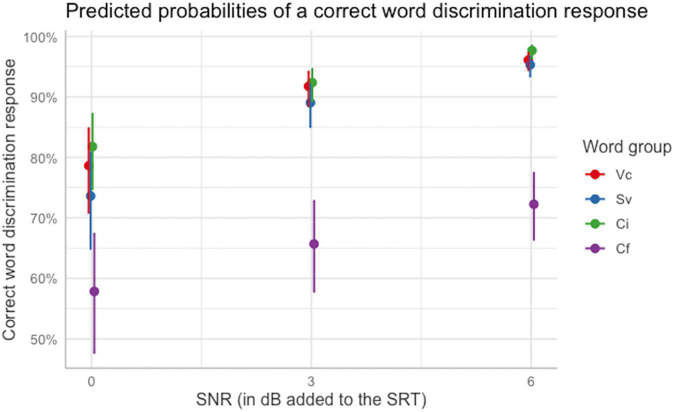
Interaction between word group and SNR. Performance for the final-consonant group was significantly lower than for other word groups across all SNRs, but performance for the simple-vowel group was significantly lower than performance for initial consonant for the lowest SNR only, accounting for a significant interaction between word group and SNR. Error bars indicate 95% confidence intervals. Plot obtained using the sjPlot package (Lüdecke, 2021).

*Post hoc* analysis (Table 5) was performed to compare performance across azimuths, SNRs, and word groups using the *ghlt* function within the *multcomp* package (Hothorn et al., 2008), and Bonferroni corrections were applied to account for repeated testing. Comparing average performance for different azimuths across SNRs and word groups, indicated that the likelihood of a correct word-discrimination response was significantly higher at the left most location, −90°, than at −30° and at the midline. Additionally, performance at the midline was significantly lower than at −60° and 60°. Performance at −60° was also significantly higher than at −30°. These outcomes further support the hypothesis of a *U*-shape for performance as a function of azimuth. No other comparisons were statistically significant. Exploration of the significant interaction (Table 6) between SNR and word group via pairwise comparisons across averaged variable levels indicated that, although there was an overall increase in performance with increasing SNR, performance for the final-consonant group was significantly lower than that for each of the other discrimination groups at each of the SNRs tested. Additionally, performance for the initial-consonant group was significantly higher than that for the simple-vowel group only at the lowest SNR. No other comparisons of word groups within each SNR were statistically significant. Figure 8 shows the predicted probabilities for each word group as a function of the SNR at which testing was conducted.

**TABLE 5:** *Post hoc* analysis for the effect of azimuth on word discrimination.

Azimuth				
**Comparison**	**Estimate**	**Std. Error**	***z*-value**	**Pr(> | z|)**
−90° vs. −60°	0.04	0.11	0.38	1.000
−90° vs. −30°	0.35	0.10	3.35	**0.017**
−90° vs. 0°	0.47	0.10	4.50	**<0.001**
−90° vs. 30°	0.25	0.11	2.38	0.367
−90° vs. 60°	0.19	0.11	1.82	1.000
−90° vs. 90°	0.28	0.12	2.37	0.372
−60° vs. −30°	0.31	0.08	3.69	**0.005**
−60° vs. 0°	0.43	0.08	5.14	**<0.001**
−60° vs. 30°	0.21	0.08	2.47	0.284
−60° vs. 60°	0.15	0.09	1.78	1.000
−60° vs. 90°	0.24	0.10	2.38	0.365
−30° vs. 0°	0.12	0.08	1.46	1.000
−30° vs. 30°	−0.10	0.08	−1.23	1.000
−30° vs. 60°	−0.16	0.08	−1.92	1.000
−30° vs. 90°	−0.07	0.10	−0.67	1.000
0° vs. 30°	−0.22	0.08	−2.69	0.150
0° vs. 60°	−0.28	0.08	−3.38	**0.015**
0° vs. 90°	−0.18	0.10	−1.85	1.000
30° vs. 60°	−0.06	0.08	−0.70	1.000
30° vs. 90°	0.03	0.10	0.34	1.000
60° vs. 90°	0.09	0.10	0.91	1.000

*P-values were Bonferroni corrected. Bold values correspond to statistically significant outcomes.*

**TABLE 6:** *Post hoc* analysis of the interaction between SNR and Word Group.

SNR	Comparison	Estimate	Std. Error	*z*-value	Pr(> | z|)
SRT	V_c_ vs. V_s_	0.28	0.10	2.80	0.342
	V_c_ vs. C_i_	−0.20	0.10	−1.91	1.000
	V_c_ vs. C_f_	0.99	0.10	10.29	**<0.001**
	V_s_ vs. C_i_	−0.48	0.10	−4.69	**<0.001**
	V_s_ vs. C_f_	0.71	0.09	7.65	**<0.001**
	C_i_ vs. C_f_	1.19	0.10	12.05	**<0.001**
SRT + 3 dB	V_c_ vs. V_s_	0.31	0.14	2.27	1.000
	V_c_ vs. C_i_	−0.08	0.15	−0.57	1.000
	V_c_ vs. C_f_	1.76	0.12	14.46	**<0.001**
	V_s_ vs. C_i_	−0.40	0.14	−2.84	0.296
	V_s_ vs. C_f_	1.45	0.11	12.95	**<0.001**
	C_i_ vs. C_f_	1.84	0.12	14.91	**<0.001**
SRT + 6 dB	V_c_ vs. V_s_	0.20	0.20	0.99	1.000
	V_c_ vs. C_i_	−0.53	0.24	−2.24	1.000
	V_c_ vs. C_f_	2.24	0.16	14.05	**<0.001**
	V_s_ vs. C_i_	−0.72	0.23	−3.16	0.106
	V_s_ vs. C_f_	2.05	0.15	13.67	**<0.001**
	C_i_ vs. C_f_	2.77	0.20	14.06	**<0.001**

*P-values were Bonferroni corrected. Comparisons are reported in this table only for pairs of word groups at each SNR. Bold values correspond to statistically significant outcomes.*

#### Comparison With Data Previously Collected Using Loudspeakers

**FIGURE 9:**
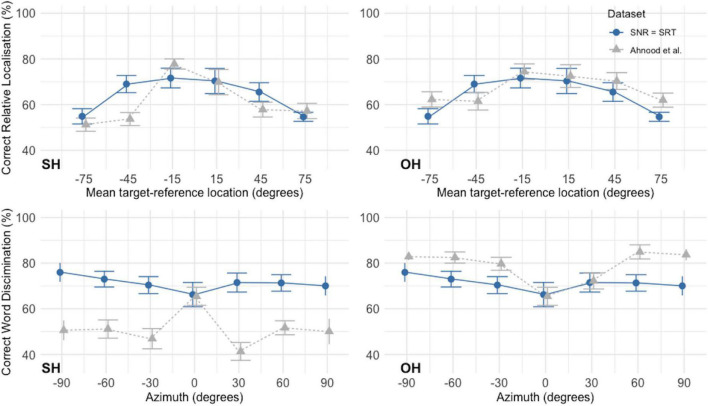
Comparison of the outcomes reported here for SNR = SRT with the outcomes obtained by Ahnood (2017) for normal-hearing participants using a loudspeaker setup. The outcomes of the present study are displayed by blue circles joined by continuous line. Ahnood’s results are displayed by gray triangles joined with dotted line. Error bars indicate standard errors. Because Ahnood, unlike us, used an asymmetric noise configuration, our data are compared separately with Ahnood’s outcomes for trials where the noise sources were located on the same hemispace than the source of the speech (SH, noise in the same hemispace), and for trials where the noise was located on the hemispace opposite to the speech (OH, noise in the opposite hemispace). Outcomes for word discrimination at the midline are plotted on both panels and were not separated by noise location. Figure made using the packages ggplot2 (Wickham, 2016) and cowplot (Wilke, 2020) in R (R Core Team, 2021).

Figure 9 shows a graphical comparison of the data collected in this study for SNR = SRT with that collected by Ahnood (2017). The SSiN-VA data is plotted separately against the responses of Ahnood’s participants for trials in which the noise sources were located in the same hemispace than the speech and for those in which the noise sources were located in the opposite hemispace than that of the speech. For relative localization, performance is quite close across datasets for both comparisons (same hemispace or opposite hemispace). For Ahnood’s dataset, there is a trend toward relative localization performance to be better at the most lateral locations when the noise was delivered from the opposite hemispace with respect to the speech, but overall, both curves are quite close to the data collected using SSiN-VA. For word discrimination, Ahnood’s outcomes vary considerably depending on whether the noise was located on the same hemispace than the speech or on the opposite hemispace. This is expected as locating the noise on the opposite hemispace would have maximized the distance between the speech and the noise sources, which in turn would have had an impact on the effective SNR at each ear. This would have increased the SRM achieved by the participants, improving word-discrimination outcomes. For the trials where Ahnood delivered the nose from the same hemispace as the speech, her participants performed better than those using SSiN-VA at the most lateral locations. For the trials where Ahnood delivered the noise from the same hemispace as the speech, her participants performed much worse than those tested here, except at the midline. These patterns are likely to be largely accounted for by the spatial separation of the speech and the noise, which was different across datasets, rather than by differences related to the virtual nature of the stimuli used here.

## Discussion

The aim of this study was to determine if the patterns of responses obtained with the SSiN-VA are similar to those obtained with loudspeaker implementations. Localization performance was predicted by mean location, SNR, and the direction of the shift from reference to target. For word discrimination, performance was predicted by azimuth, SNR, and word group and a significant interaction between word group and SNR was found. In what follows these results are discussed in more detail.

### Shape of the Performance Functions

As for loudspeaker data, relative localization followed the pattern of an inverted-*U* shape and word discrimination followed a *U*-shaped pattern (Bizley et al., 2015; Ahnood, 2017), confirming that SSiN-VA leads to patterns of performance similar to those previously found with loudspeaker implementations. This makes sense, because as azimuth (or mean location) is varied, the availability of cues for each task, relative localization and word discrimination, changes. The relative levels of the signals arriving at each ear (ILDs) and their relative timing (ITDs) increases the further away the sources are from the midline. Additionally, the relative SNRs across ears change. In other words, the availability of binaural cues and binaural effects such as binaural summation or binaural squelch varies across spatial locations.

The inverted *U*-shape pattern of our data reproduces the outcomes found by Bizley et al. (2015) and Ahnood (2017) using the same stimuli, albeit with a different spatial location of the noise sources. As pointed out by Bizley et al. (2015), this pattern was also observed in a previous study where broadband noise was used (Wood and Bizley, 2015). In the same study, as well as in previous work (Butler, 1986), using spectrally restricted stimuli led to a more marked decrease in performance at the most lateral spatial locations. The improvement of relative localization performance around the midline is consistent with the idea that, because ILDs are roughly proportional to the sine of the azimuthal angle, horizontal localization errors should increase monotonically with increasing azimuth (Middlebrooks and Green, 1991). This is similar to the outcomes reported by Bizley et al. (2015) for one of their experiments in which they used loudspeakers separated at 15° intervals, although these authors did not find a significant effect of azimuth on relative-localization performance for another experiment where they used a 30°-interval separation for most of their loudspeakers. However, the type of analysis carried out here is different to that carried out by Bizley et al. (2015), and there may be power differences across studies underlying the discrepancy.

The *U*-shaped pattern observed for word discrimination is also consistent with the outcomes obtained with the original implementation of the SSiN (Bizley et al., 2015), and with other published work. For example, Laitakari and Laitakari (1997) also reported improved speech discrimination at 90°compared to 0°. In conditions where the background noise is symmetrically distributed around the midline, the advantage of lateral locations with respect to the midline for speech discrimination may arise from factors such as the “better-ear glimpsing” effect (Glyde et al., 2013), where information from the ear with better SNR is used to make sense of the speech. This difference in SNR across ears is partly underpinned by the head-shadow effect. Thus, this effect is likely to help to improve performance for speech sources that are away from the midline. ITDs are likely to contribute too, as they can be used to achieve binaural unmasking of the low-frequency portions of a signal (Hawley et al., 2004).

In spite of our data having the expected shape in terms of performance as a function of spatial location, there were some asymmetries, i.e., performance to the left and the right of the midline was sometimes significantly different. This trend is also apparent in loudspeaker data such as that reported by Bizley et al. (2015) and Ahnood (2017). It is possible that this is due to noise in the data arising from individual performance. This should be taken into account when interpreting clinical outcomes.

Overall, these results are encouraging and suggest that the use of this virtual implementation of the SSiN leads to similar patterns of responses to loudspeaker implementations. The next steps in the development of the prototype are to manipulate different parameters in order to achieve varying levels of difficulty. This is key to the clinical implementation of SSiN-VA, as the test is conceived as a flexible tool able to test a wide range of clinical populations with diverse spatial-listening skills.

### Effect of the Signal-to-Noise Ratio on Each of the Tasks

Unlike previous work with the SSiN test, the effect of SNR on performance was measured. SNR had a strong effect on speech-discrimination performance, with each 3-dB increase in SNR leading to a significant improvement in word recognition. However, this effect should not be interpreted in isolation, as there was an interaction between SNR and word group (this will be discussed below). The impact of SNR on relative localization was lower, with a trend toward improving performance with increasing SNR, but where a 6-dB increase in SNR above the SRT led to significantly improved performance, a 3-dB increase did not. The greater impact of SNR on word recognition than on relative localization may have arisen from the fact that speech discrimination requires the audibility of specific parts of the two speech signals. There would have been instances where audibility was appropriate for detection but not for discrimination. Conversely, relative localization is still possible even if audibility is not enough for discrimination.

### Effect of Word Group on Each of the Tasks

Contrary to Bizley et al. (2015), who reported that relative localization was better for the final-consonant group, no effect of word group on relative-localization performance was found. However, there was a trend in the same direction in our data which did not reach statistical significance. Bizley et al. (2015) proposed that the listener, knowing that the discrimination cue is at the end, has more time to listen for the localization cues before focusing on the discrimination cues.

There was a significant effect of word group for the word-discrimination task, also in contrast with the findings of Bizley et al. (2015). Here, the effect of word group on word discrimination can be largely accounted for by the much lower likelihood of a correct response for the final-consonant group at each SNR. The consonants in this group were plosive and fricative consonants, characterized by their predominantly high-frequency energy. These phonemes might be more vulnerable to being masked by the babble noise, depending on the babble level. For example, the relative amplitude of the formant transition F3 of the voiced fricative /z/ (as in “cheese”) with respect to the adjacent vowel is about −16.3 dB (Jongman et al., 2000). The other consonants in the final-consonant group were the voiceless stops /p/, /t/, /k/. These phonemes are characterized by a brief silence followed by a brief burst, which is an important discrimination cue (Kapoor and Allen, 2012). This burst would have been susceptible to being easily masked by the background babble. Additionally, initial consonants are typically more discriminable than final consonants due to their lower level and shorter duration, and due to the higher amount of information present in the consonant-vowel formant transition compared to the vowel-consonant transition (Redford and Diehl, 1999). There was also a difference between the initial-consonant group and the simple-vowel group which was only significant at the lowest SNR, so that at the SRT condition, correct word discrimination was less likely for the simple-vowel discrimination group than for the initial-consonant group. The interaction of SNR with word group suggests that the audibility of the cues played a role, as the simple-vowel-initial consonant difference was significant only at the lowest SNR.

In spite of the possible impact of these acoustic and perceptual differences across word groups, it cannot be ruled out that at least part of this effect was underpinned by deviations of the headphone frequency response from the free field response. This could explain the conflicting findings for the effect of word group on each of the tasks across this study and Bizley et al. (2015). The effect of the transducers will be investigated in future research, as it is necessary to be aware of any limitations imposed by the transducers before the test is generalized for clinical use.

### Direction Bias

Unexpectedly, when the target word shifted to the right of the reference word, a correct relative-localization response was more likely. Ocklenburg et al. (2010) reported that participants carrying out a sound-localization task showed a bias toward the opposite side to the dominant hand when they pointed at the source of the sound using their hand or their head. Here, participants did not point at the source of the stimuli but used a computer interface where they had buttons to click on, labeled “left” and “right” (as shown in Figure 2). Participants were not asked whether they were right- or left-handed, but it is reasonable to assume that most of them would have been right-handed. It is difficult to compare across these studies because the nature of the localization task (absolute vs. relative localization) and the mode of giving a response differed. However, our results show an effect that appears to be in conflict with what was reported in the literature. Inspection of the individual data suggested that the bias was large for one subject and much smaller for others. Three subjects showed the opposite pattern (bias to the left) and three other subjects showed very small differences across shift directions.

### Location of the Noise Sources

One of the possible parameters for adjustment in future versions of the test is the location of the noise sources. The location of the noise sources has an impact on the shape of the performance function, especially for word discrimination. This is evident from Figure 9, which compares the data collected here with Ahnood’s dataset. For Ahnood’s dataset, speech discrimination was strongly affected by the location of the noise, as speech discrimination is highest when the spatial separation between speech source and babble noise is maximized, and vice versa (Hirsh, 1950). Thus, Ahnood’s participants’ responses were expected to show greater differences across spatial locations compared to our participants as, in the case of the latter, symmetrical maskers were used. Our participants would have been more reliant on “glimpsing” (Glyde et al., 2013), i.e., on extracting information during short-term improvements in SNR which, with symmetrical maskers, will occur alternatively at one ear or the other (Brungart and Iyer, 2012). The differences in patterns of response across the datasets should be more evident for the comparison between word-discrimination functions than for the comparison between relative-localization functions, as localization performance is relatively independent from source-masker spatial separation. Figure 9 supports these predictions. Ahnood’s noise configuration was shown to lead to different patterns of responses for normal-hearing participants and cochlear-implant users (Ahnood, 2017). The potentially large separations between speech and babble source are better suited to test SRM but would have required us to increase the number of trials in our test. As the purpose of this work was to determine if the virtual implementation led to similar patterns of responses than the original loudspeaker implementation, a symmetric configuration was used, similarly to Bizley et al. (2015), although the number of sources was reduced. Having a symmetrical configuration allowed the use of a simpler design. Moving forward toward a clinical implementation of the test, a direct comparison of a loudspeaker setup and this virtual implementation using different noise configurations (symmetric and asymmetric) is due to be carried out.

### Limitations of the Auralization Technique

Auralization using HRTFs that are not individualized may lead to inaccurate sound localization and issues with externalization (Stitt et al., 2019). Furthermore, some training might be needed in order to achieve performance at similar levels than with individualized HRTFs (Blum et al., 2004; Parseihian and Katz, 2012; Steadman et al., 2019; Stitt et al., 2019). HRTFs include a spectral component which is used for front-back judgments and localization along the vertical axis, and an interaural component given by the ITDs and ILDs. Here the size of the head of each participant was used to personalize ITDs. Stitt et al. (2019) found that, even when head-circumference-based ITDs are used, the localization performance error increases to 15.5°–19.4° from the 9.3°–12.5° measured from a control group using individual HRTFs. Parseihian and Katz (2012) reported an increase from 13°–16° to 17°–25° between a group with individualized HRTFs and groups with non-individualized HRTFs. Training using a VR videogame (Steadman et al., 2019) or providing proprioceptive feedback (Parseihian and Katz, 2012; Stitt et al., 2019) did not significantly improve lateral angle judgments. The relative-localization task performed here requires spatial discrimination with 30° resolution. As this is generally larger than the average errors encountered by these investigators, it is likely that performance would have been similar with individualized HRTFs. However, there may be individual cases where the introduced error makes it hard to give a relative-localization response. Informal feedback given by a few participants during the task was consistent with some front-back confusions and with reports of the two words originating from the same source. The impact of using non-individualized HRTFs with participants who have hearing loss should be investigated.

### Limitations of the Study

A small sample size of 12 participants with normal hearing was used. This is similar to previous studies with loudspeakers (Bizley et al., 2015; Ahnood, 2017). Testing a larger sample of participants including examining the effects of age, hearing status, and co-existence of other disabilities on the user experience with virtual audio might be of interest in order to optimize SSiN-VA for a wide range of users.

Other limitations of the present study are that all data were collected with SSiN-VA and that no data were obtained with a loudspeaker setup, and that the existing dataset used for comparison was generated using a noise-location configuration different than that used here. As explained in the Results section, this should lead to some differences in the patterns of responses, especially for the speech-discrimination task. Further work with SSiN-VA will address this issue by directly comparing both setups using the same noise configuration.

## Conclusion

The findings reported in this study support the use of virtual-audio to develop clinical-audiology applications to assess spatial listening skills. The SSiN-VA led to similar patterns of responses than SSiN for speech discrimination and relative localization as a function of the spatial location of the sound sources. This suggests that binaural spatialization has the potential to make a step change in the clinical testing of spatial hearing abilities, making it possible to inexpensively assess the benefits of different hearing devices, such as bilateral hearing aids, bilateral or bimodal cochlear implants, and devices used to help people with unilateral hearing loss or single-sided deafness. This approach also increases clinical efficiency because testing can be carried out in the home if necessary.

Simplifying the equipment and space requirements to conduct reliable tests that assess complex listening skills, including the development of home-testing versions, ultimately increases the equality of access to hearing care across geographical location and improves the quality of care, enhancing the experience of the patients and their families.

## Data Availability Statement

The datasets presented in this study can be found in Apollo, the online repository of the University of Cambridge, following the link: 10.17863/CAM.76227.

## Ethics Statement

The studies involving human participants were reviewed and approved by Cambridge Psychology Research Ethics Committee (Ref. 2019.093) and Joint Research Compliance Office at Imperial College (Ref. 19IC5073). The participants provided their written informed consent to participate in this study.

## Author Contributions

MS-C, LP, and DV designed the experiments. MS-C collected the data and wrote the first draft of the manuscript. LP developed all versions of the SSiN-VA app. BW and MS-C calibrated the speech stimuli. All authors contributed to the analysis of results and to the writing of the manuscript.

## Author Disclaimer

The views expressed are those of the authors and not necessarily those of the NIHR or the Department of Health and Social Care.

## Conflict of Interest

The authors declare that the research was conducted in the absence of any commercial or financial relationships that could be construed as a potential conflict of interest.

## Publisher’s Note

All claims expressed in this article are solely those of the authors and do not necessarily represent those of their affiliated organizations, or those of the publisher, the editors and the reviewers. Any product that may be evaluated in this article, or claim that may be made by its manufacturer, is not guaranteed or endorsed by the publisher.
